# Pangenomic and functional domain comparison of enterotoxigenic *Escherichia coli* isolated from humans and swine: insights into host specificity

**DOI:** 10.1007/s00438-026-02412-4

**Published:** 2026-04-24

**Authors:** Gabriela Merker Breyer, Juliana Silva Bernardes, Márcio Dorn, Franciele Maboni Siqueira

**Affiliations:** 1 Laboratório de Bacteriologia Veterinária (LaBacVet), Departamento de Patologia Veterinária, Universidade Federal do Rio Grande do Sul, Porto Alegre, Brazil; 2 Programa de Pós-Graduação em Ciências Veterinárias, Universidade Federal do Rio Grande do Sul, Porto Alegre, Brazil; 3 Station Biologique de Roscoff, Roscoff, France; 4 Centro de Biotecnologia (CBiot), Universidade Federal do Rio Grande do Sul, Porto Alegre, Brazil; 5 Instituto de Informática, Universidade Federal do Rio Grande do Sul, Porto Alegre, Brazil

**Keywords:** ETEC, Host specificity, Functional domains, Pangenome analysis

## Abstract

Diarrhea caused by enterotoxigenic *Escherichia coli* (ETEC) is a common threat to humans and animals. Clinical ETEC strains display host tropism, usually driven by adherence and toxin-host interactions in the gut. Other virulence factors, metal acquisition mechanisms, and immune evasion strategies may also influence host specificity. Using publicly available genomes, we performed a pangenomic and functional comparison of 77 human- and swine-derived ETEC strains. Sequence types ST10 and ST4 found in both hosts, suggesting potential cross-host transmission. Phylogenetic analyses showed clustering mainly by ST, regardless of host or geography. Additionally, most functional domains were shared between hosts; however, human-derived strains carried exclusive domains related to adhesion, transposition, and toxins, whereas swine-derived strains harbored domains linked to stress response and metal binding. Notably, the PF09075 domain from the STb enterotoxin occurred only in swine-derived strains, despite previous reports in humans. Comparative analyses of STa (PF02048) and STb revealed no structural or functional homology but confirmed the swine-specific association of STb and greater sequence STa variability in human-derived strains. These findings indicate that sequence and functional differences in these enterotoxins contribute to ETEC host tropism, while ST patterns reveal possible epidemiological links between human and swine infections.

**Supplementary Information:**

The online version contains supplementary material available at 10.1007/s00438-026-02412-4.

## Introduction

Diarrhea caused by enterotoxigenic *Escherichia coli* (ETEC) is a threat to both humans and animals (Vipin Madhavan and Sakellaris 2015; Dubreuil et al. 2016). In humans, most clinical reports describe mild infections in infants and travelers, commonly occurring in low-income countries with poor sanitation infrastructure. In animals, ETEC infections are a significant economic concern in livestock production, mainly in the swine industry, causing high mortality rates associated with neonatal colibacillosis and post-weaning diarrhea (Fairbrother et al. 2005).

ETEC strains appear to be host-specific, likely due to microbial adherence to specific receptors in the host’s gut, as well as interactions between ETEC toxins and host receptors after colonization (Dubreuil 2012; Baumler and Fang 2013; Vipin Madhavan and Sakellaris 2015). Nevertheless, other genomic features may contribute to host specificity, including metal acquisition mechanisms, evasion of the host immune system, replication efficiency, horizontal gene transfer, genome arrangements, and genomic decay (Baumler and Fang 2013). Although colonization factors and toxin-receptor interactions are recognized as primary drivers of host tropism, a systematic genome-wide comparison of additional functional elements potentially associated with host adaptation has not yet been comprehensively performed for human versus swine ETEC strains.

Comparative pangenome analysis based on whole-genome data may help identify genetic features related to host specificity. Previous pangenomic studies have explored genomic differences between commensal and pathogenic *E. coli* (Rasko et al. 2008), but no such studies have focused specifically on the ETEC pathotype. Therefore, this study compares the functional domains, defined as conserved protein regions associated with specific molecular functions and molecular roles, of ETEC strains isolated from human and swine stool samples from different countries, aiming to explain their tropism.

## Materials and methods

### Genome sequences

We retrieved the complete genome sequences and corresponding protein data of 77 ETEC strains isolated from human (n = 47) and swine (n = 30) stool samples, available in the NCBI database in September 2023 (Table 1). Sequence type information for the analyzed strains was further obtained using multilocus sequence typing (MLST-Achtman) based on the PubMLST database (Jolley et al. 2018).

**Table 1:** Description of enterotoxigenic *Escherichia coli* strains isolated from human and swine hosts

ID	NCBI genome assembly	Host	Serotype	ST	Date	Location	Sequencing plataform
H1	ASM21047v1	Human	O78:H11:K80	48	< 1973	Bangladesh	nd
H2	ASM2458502v1	Human	O109:H21	155	1979	Philippines	Illumina MiSeq
H3	ASM2458500v1	Human	O71:H7	1308	1984	Thailand	Illumina MiSeq
H4	ASM2458488v1	Human	O1:H8	297	1979	Brazil	Illumina MiSeq
H5	ASM1337689v1	Human	nd	11,231	1986	Bangladesh	Illumina MiSeq
H6	ASM2458494v1	Human	O112:H21	6160	1979	Brazil	Illumina MiSeq
H7	ASM2458484v1	Human	nd	6402	1984	Nigeria	Illumina MiSeq
H8	ASM2458492v1	Human	nd	2521	1985	Thailand	Illumina MiSeq
H9	ASM188807v1	Human	O6:H16	4	1987	Mexico	Illumina HiSeq + PacBio RSII
H10	ASM230231v1	Human	nd	443	2006	Bangladesh	PacBio
H11	ASM230233v1	Human	nd	5305	2006	Bangladesh	PacBio
H12	ASM395624v1	Human	nd	398	1974	Mexico	PacBio
H13	ASM395632v1	Human	O37	443	2010	Gambia	PacBio
H14	ASM395648v1	Human	nd	443	2012	Gambia	PacBio
H15	ASM395616v1	Human	nd	173	2010	India	PacBio
H16	ASM395620v1	Human	nd	443	2010	Mali	PacBio
H17	ASM395636v1	Human	O117:HNT	443	2009	Bangladesh	PacBio
H18	ASM395618v1	Human	O115	443	2010	India	PacBio
H19	ASM395644v1	Human	O1/O148:H28	nd	2009	India	PacBio
H20	ASM395640v1	Human	O128	2332	2010	India	PacBio
H21	ASM395642v1	Human	O148	94	2010	India	PacBio
H22	ASM395622v1	Human	O25	1312	2010	Mali	PacBio
H23	ASM395628v1	Human	O148	94	2010	India	PacBio
H24	ASM395626v1	Human	nd	1201	2012	India	PacBio
H25	ASM395630v1	Human	O128	10	2008	Mozambique	PacBio
H26	ASM395638v1	Human	O8	517	2008	Bangladesh	PacBio
H27	ASM395634v1	Human	nd	1564	2012	Pakistan	PacBio
H28	ASM1643280v1	Human	O8:H9	410	2018	Ghana	Illumina MiSeq + Oxford Nanopore MinION
H29	ASM335138v1	Human	O8:H21	410	2017	United Kingdom	Oxford Nanopore MiniION; + Illumina HiSeq
H30	ASM1888438v1	Human	O115:H5	443	1997	Guinea-Bissau	PacBio
H31	ASM1888434v1	Human	nd	173	nd	Bangladesh	PacBio
H32	ASM1888454v1	Human	O8:H9	423	1990	Saudi Arabia	PacBio
H33	ASM1888448v1	Human	nd	4121	nd	Chile	PacBio
H34	ASM1888458v1	Human	O8:H-	88	2003	Turkey	PacBio
H35	ASM2237667v1	Human	nd	2332	nd	Saudi Arabia	PacBio
H36	ASM1888428v1	Human	O166:H27	58	nd	South Africa	PacBio
H37	ASM1888430v1	Human	nd	223	nd	Egypt	PacBio
H38	ASM1888436v1	Human	nd	2332	nd	Egypt	PacBio
H39	ASM1888442v1	Human	nd	278	nd	nd	PacBio
H40	ASM1888450v1	Human	O25:H-	1312	nd	nd	PacBio
H41	ASM1888440v1	Human	O6:H16	4	1997	Guinea-Bissau	PacBio
H42	ASM1888446v1	Human	nd	410	nd	Mexico	PacBio
H43	ASM1888432v1	Human	nd	10	nd	Saudi Arabia	PacBio
H44	ASM1888452v1	Human	O6:H16	4	1971	Vietnam	PacBio
H45	ASM1888456v1	Human	O114:H-	2368	1993	Egypt	PacBio
H46	ASM1888444v1	Human	O19:H45	728	nd	Guinea-Bissau	PacBio
H47	ASM837533v1	Human	nd	48	2016	Bangladesh	Illumina MiSeq + Oxford Nanopore MiniION
S1	ASM280380v2	Swine	O147:K89	764	2016	China	PacBio
S2	ASM289390v2	Swine	O9:K103	90	2016	China	PacBio
S3	ASM21271v2	Swine	O149	165	2007	USA	Roche 454
S4	ASM22000v2	Swine	O147	10	2007	USA	Roche 454
S5	ASM2466225v1	Swine	nd	10	2018	China	Illumina NovaSeq
S6	ASM3001359v1	Swine	nd	42	2012	Belgium	Oxford Nanopore GridION
S7	ASM3001310v1	Swine	nd	5786	2012	Belgium	Oxford Nanopore GridION
S8	ASM3001343v1	Swine	nd	100	2021	Belgium	Oxford Nanopore GridION
S9	ASM3001371v1	Swine	nd	10	2021	Belgium	Oxford Nanopore GridION
S10	ASM3001367v1	Swine	nd	10	2021	Belgium	Oxford Nanopore GridION
S11	ASM3001347v1	Swine	nd	100	2021	Belgium	Oxford Nanopore GridION
S12	ASM3001316v1	Swine	nd	100	2012	Belgium	Oxford Nanopore GridION
S13	ASM3001300v1	Swine	nd	2074	2021	Netherlands	Oxford Nanopore GridION
S14	ASM3001266v1	Swine	nd	10	2020	Belgium	Oxford Nanopore GridION
S15	ASM3001378v1	Swine	nd	nd	2020	Belgium	Oxford Nanopore GridION
S16	ASM3001324v1	Swine	nd	993	2021	Belgium	Oxford Nanopore GridION
S17	ASM3001373v1	Swine	nd	90	2020	Belgium	Oxford Nanopore GridION
S18	ASM3001349v1	Swine	nd	993	2021	Belgium	Oxford Nanopore GridION
S19	ASM3001384v1	Swine	nd	nd	2020	Belgium	Oxford Nanopore GridION
S20	ASM3001357v1	Swine	nd	772	2021	Belgium	Oxford Nanopore GridION
S21	ASM3001276v1	Swine	nd	744	2012	Belgium	Oxford Nanopore GridION
S22	ASM3001386v1	Swine	nd	772	2020	Belgium	Oxford Nanopore GridION
S23	ASM3001382v1	Swine	nd	772	2020	Belgium	Oxford Nanopore GridION
S24	ASM3001380v1	Swine	nd	10	2020	Belgium	Oxford Nanopore GridION
S25	ASM3001353v1	Swine	nd	10	2021	Belgium	Nanopore
S26	ASM3001355v1	Swine	nd	10	2021	Belgium	Nanopore
S27	ASM3001351v1	Swine	nd	nd	2021	Belgium	Nanopore
S28	ASM3001369v1	Swine	nd	nd	2021	Belgium	Nanopore
S29	ASM3001365v1	Swine	nd	nd	2021	Belgium	Nanopore
S30	ASM3001268v1	Swine	nd	772	2020	Belgium	Nanopore

*ST* sequence type, *nd* information not described

^*^Number of proteins according to the National Center for Biotechnology Information (NCBI)

### Pangenomic and phylogenetic analyses

We initially performed a pangenome analysis using Roary software (Page et al. 2015) based on annotations generated by Prokka (Seemann 2014), to identify the core and accessory genes of the analyzed ETEC strains, and to assess their pangenome accumulation curve. Maximum-likelihood phylogenetic trees for the human- and swine-derived ETEC were built with MEGA11 (Tamura et al. 2021) (bootstrap = 1000), using the core and accessory genome alignments provided by Roary.

Additionally, to assess the clonality of the analyzed genomes, single-nucleotide polymorphism (SNP) trees were built for human- and swine-derived ETEC, separately. Briefly, SNP extraction was performed by snp-sites (Page et al. 2016) based on the core genome alignments of each group, and a heatmap was built using pheatmap (Kolde R 2025) to determine SNP distribution across the genomes (scale 0 to > 8,000 SNPs).

### Phylogenetic orthology inference, domain annotation and architecture

ETEC phylogenetic orthology was inferred using OrthoFinder (Emms and Kelly 2019), and orthogroups and proteins exclusive to human or swine isolates were identified.

In parallel, protein domains were predicted by MyCLADE software (Vicedomini et al. 2021) with the Pfam database (Mistry et al. 2021) and domain architectures were reconstructed by DAMA (default parameters) (Bernardes et al. 2016). We assessed the 50 most frequent ETEC domains predicted in human and swine strains, as well as shared and host-exclusive domains. A Venn diagram was constructed using ggvenn package (Yan 2023) in RStudio (RStudio Team 2015).

Biological functional information for shared and exclusive domains was retrieved from Pfam and UniProt (Bateman et al. 2023). Domains annotated as putative, uncharacterized, or of unknown function in bacteria (according to Pfam), or in the *Escherichia* taxon [562] (according to UniProt), were classified as “unknown function”. Statistical differences in Pfam domain frequencies between hosts were calculated using t-test (*p* < 0.05) and visualized with the ggpubr package (Kassambara 2023). Host-associated domains were determined based on their presence or absence patterns across isolates from each host, rather than formal gene-trait association testing. Only exclusive domains present in at least 25% of genomes from each host were considered for further analyses.

Domain architecture analysis of the host-exclusive proteins containing unique domains was performed using DAVI (Bernardes et al. 2016), employing DAMA with hierarchical clustering. We then compared the domain of proteins containing host-exclusive domains across human and swine ETEC strains to evaluate whether such structural differences could explain host specificity.

### Similarity analysis of heat-stable enterotoxins from ETEC isolated from swine and humans

To investigate host-specific features of heat-stable enterotoxins in ETEC, we focused on: i) PF09075 (STb secrete) domain present in heat-stable enterotoxin II (STb, UniProt ID: P22542); and ii) PF02048 (enterotoxin ST) domain harbored by heat-stable enterotoxin I (STa, UniProt ID: P01559). A set of protein sequences was clustered based on similarity to generate representative consensus sequences for each group. Sequences were obtained from a FASTA file with protein sequences and processed using the BioPython library (SeqIO). Pairwise sequence identity was calculated through a direct positional alignment without gaps. Similarity was determined by the ratio of identical residues to the minimum alignment length, multiplied by 100. These identity values were stored in a symmetric matrix, enabling comprehensive sequence comparisons. The identity matrix was used to construct a dendrogram using average linkage clustering method, and a cutoff threshold of 80% to define clusters, grouping sequences with high similarity. For each cluster, a consensus sequence was generated by identifying the most frequent residue at each aligned position. Additionally, a heatmap was generated using the Seaborn library to visualize the identity matrix and reveal similarity patterns among sequences. All analyses were performed in Python employing BioPython for sequence handling, NumPy for matrix calculations, SciPy for hierarchical clustering, and Matplotlib/Seaborn for visualization. Residue counting was performed using Collections.Counter. Structural alignment of 7D37 and AF-A0A193M1P5-F1-v4 was performed using PyMOL, resulting in a root-mean-square deviation (RMSD) of 5.943 Å. The three-dimensional (3D) structures for the three studied protein sequence clusters were modeled using the AlphaFold server.

## Results

### Phylogenetic relationship of enterotoxigenic *Escherichia coli* from human and swine

MLST analysis identified 39 distinct sequence types (STs) among the analyzed ETEC strains available in PubMLST, while six swine-derived ETEC strains could not be matched to any existing STs in the current database (Table 1). Although the dataset size is limited, most STs appeared to be host-specific, with the exception of *E. coli* ST10 and *E. coli* ST4 that were found in both human and swine isolated. Among the human-derived ETEC strains, *E. coli* ST443 were the most frequent (7/41); whereas in swine, *E. coli* ST10 (8/36) and ST772 (4/36) were the most common.

**Fig. 1:**
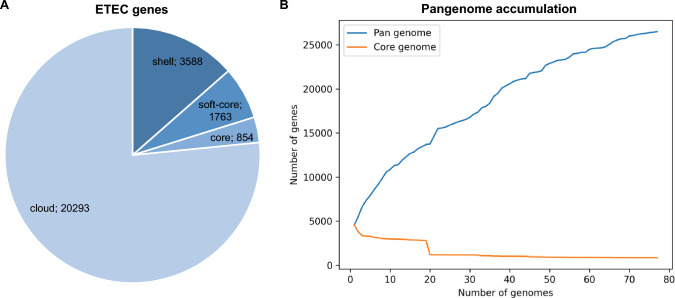
Roary pangenome statistics of human- and swine-derived enterotoxigenic *Escherichia coli*. **A** Pie chart of gene distribution in core (in ≥ 99% of the analyzed genomes), soft-core (95–99%), shell (15–95%), and cloud (≤ 15%) genome. **B** Pangenome accumulation curve of ETEC genes in the analyzed genome dataset

**Fig. 2:**
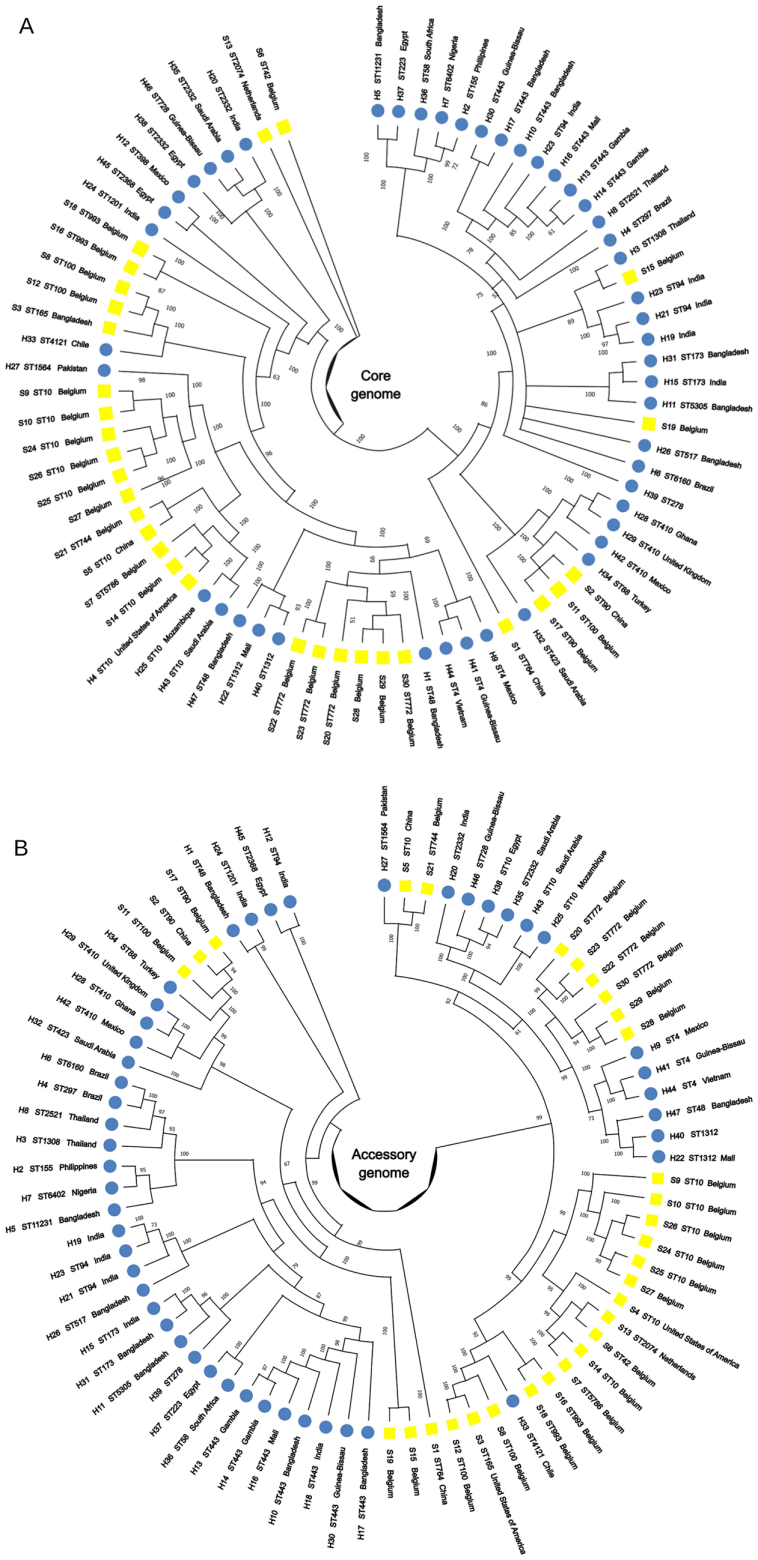
Phylogenetic analyses of enterotoxigenic *Escherichia coli* strains from human (blue) and swine (yellow) hosts. Phylogenetic trees were built based on: **A** core genome, and **B** accessory genome alignments. Bootstrap frequencies are indicated at each branch (cutoff = 50)

Pangenome analysis of the 77 ETEC genomes included in this study revealed an open and highly dynamic genome structure, with 26,498 genes. The strict core genome comprises only 854 genes, whereas a vast accessory genome (cloud fraction of 20,293 genes) (Fig. 1A). Accordingly, the accumulation curves showed the increase of the pangenome and the reduction of the core genome as genomes were sequentially added (Fig. 1B). Phylogenetic analyses based on core and accessory genomes showed no clear clustering by host species; however, we observed that ETEC strains sharing the same ST were closely related in both phylogenetic trees, regardless of their geographical location (Fig. 2). In the core genome tree, swine ETEC S6 (ST2074—Netherlands) and S13 (ST42—Belgium) were distantly related to the other strains (Fig. 2A). While in the accessory genome tree, human ETEC strains H1 (ST48 – Bangladesh), H24 (ST1201—India), H45 (ST2368—Egypt), and H12 (ST94—India) showed greater phylogenetic distance compared to the other strains (Fig. 2B).

**Fig. 3:**
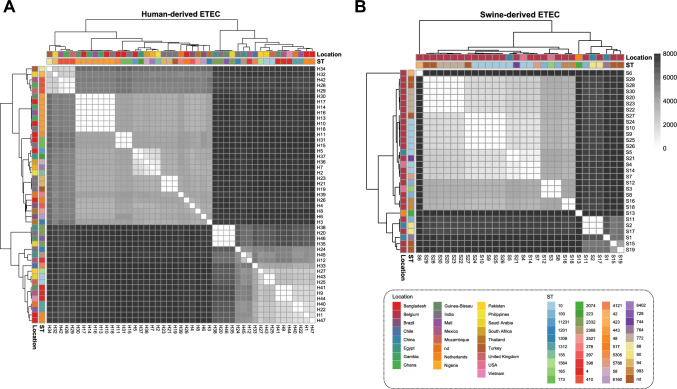
Distribution of single-nucleotide polymorphisms (SNP) in human- and swine-derived enterotoxigenic *Escherichia coli*. **A** Heatmap of the 47 ETEC genomes isolated from human. **B** Heatmap of 30 ETEC genomes isolated from swine

The SNP distribution analysis demonstrated the presence of several subclades within both human- and swine-derived ETEC populations, indicating genetic variability within the analyzed dataset (Fig. 3). In detail, human-derived ETEC genomes showed a broader distribution across the SNP distance matrix (Fig. 3A), suggesting higher genomic variability compared with swine-derived ETEC, which showed some clusters with low intra-clade SNP distances (Fig. 3B), suggesting closely related strains.

### Orthology and identification of shared and host-specific domains

**Fig. 4:**
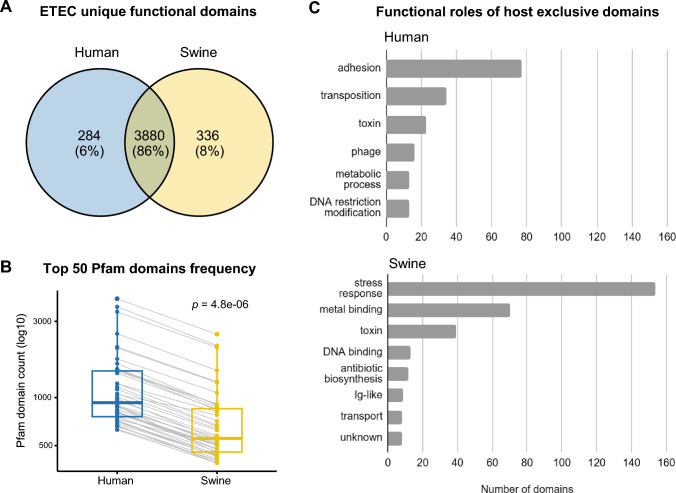
Functional domains in enterotoxigenic *Escherichia coli* isolated from human (blue) and swine (yellow) origins. **A** Venn diagram of unique Pfam domains predicted in human- and swine-derived ETEC strains. **B** Comparison of the most frequent Pfam domain counts between human- and swine-derived ETEC strains. Statistical differences were assessed using t-test (*p* < 0.05). **C** Biological function of host-specific ETEC domains from human and swine hosts. Only domains predicted in at least 25% of the ETEC strains from each respective host were included

MyClade analysis predicted a total of 608,622 functional domains across all analyzed ETEC strains, including 374,114 in human-derived strains and 234,508 in swine-derived strains (Supplementary Table 1). Of these, 4,164 and 4,216 distinct domains were identified in human and swine ETEC strains, respectively. A total of 3,880 (86%) domains were shared between both hosts, whereas 284 and 336 were exclusive to human- and swine-derived ETEC strains, respectively, considering the analyzed dataset (Fig. 4A). Additionally, domain architecture analysis revealed that most host-specific proteins carried a single domain.

In addition, we investigated the top 50 most frequent functional domains found in ETEC strains from each host and observed that all of them were shared between human- and swine-derived strains (Supplementary Table 2). The most represented domains included: PF05593, involved in ligand binding; PF13304 and PF00005, associated with ATP binding; PF00528, related to transmembrane transport; PF00126 and PF03466, involved in transcription regulation; and PF00072, related to the phosphorelay signal transduction system. Although no host-specific domains were identified among the top 50, we observed that their overall frequency was significantly higher in human-derived ETEC strains compared to those from swine (*p* < 0.05; Fig. 4B). Although the higher number of human-derived genomes (n = 47) contributes to the higher absolute frequencies observed for each domain, the differences in ranking are consistent and statistically significant, and therefore cannot be explained solely by sample size.

Host-specific domains identified in ETEC strains are detailed in Supplementary Table 3. Of these, only those found in ≥ 25% of strains within a host were considered for further biological interpretation, to focus on consistently associated features rather than rare or strain-specific events. Hence, we observed that most human-specific domains were associated with adhesion, transposition, and toxin-related functions, whereas swine-specific domains were predominantly linked to stress response, metal binding, and toxins, including the heat-labile enterotoxin (STb; PF09075) (Table 2; Fig. 4C).

**Table 2:** Most frequent host-specific functional domains in enterotoxigenic *Escherichia coli*

Human					
Pfam ID	Domain	Total count	Strains with this domain		Function
			Total	%	
PF05860	Haemagg_act	23	21	45	Toxin
PF18071	HMW1C_N	23	21	45	Adhesion
PF18254	HMw1_D2	23	21	45	Adhesion
PF05946	TcpA	17	17	36	Adhesion
PF13542	HTH_Tnp_ISL3	17	15	32	Transposition
PF14690	zf-ISL3	17	15	32	Transposition
PF05133	Phage_prot_Gp6	16	15	32	Phage
PF16831	CssAB	14	14	30	Adhesion
PF03403	PAF-AH_p_II	13	13	28	Metabolic process
PF13156	Mrr_cat_2	13	13	28	DNA restriction-modification

Swine					
Pfam ID	Domain	Total count	Strains with this domain		Function
			Total	%	
PF09075	STb_secrete	18	17	57	Toxin
PF05225	HTH_psq	13	13	43	DNA binding
PF03515	Cloacin	14	12	40	Toxin
PF02342	TerD	91	9	30	Stress response
PF12500	TRSP	14	9	30	Stress response
PF15609	PRTase_2	14	9	30	Stress response
PF06722	DUF1205	12	9	30	Antibiotic biosynthesis
PF11202	PRTase_1	12	9	30	Stress response
PF15608	PELOTA_1	12	9	30	Stress response
PF13205	Big_5	9	9	30	Immunoglobulin-like
PF00699	Urease_beta	20	8	27	Metal binding
PF15632	ATPgrasp_Ter	11	8	27	Stress response
PF00547	Urease_gamma	10	8	27	Metal binding
PF01730	UreF	10	8	27	Metal binding
PF02655	ATP-grasp_3	10	8	27	Metal binding
PF02814	UreE_N	10	8	27	Metal binding
PF05194	UreE_C	10	8	27	Metal binding
PF11570	E2R135	8	8	27	Toxin
PF12787	EcsC	8	8	27	Unknown
PF16083	Phage_holin_3_3	8	8	27	Transport

### Sequence and structural divergence of heat-stable enterotoxins in swine and human ETEC strains

**Fig. 5:**
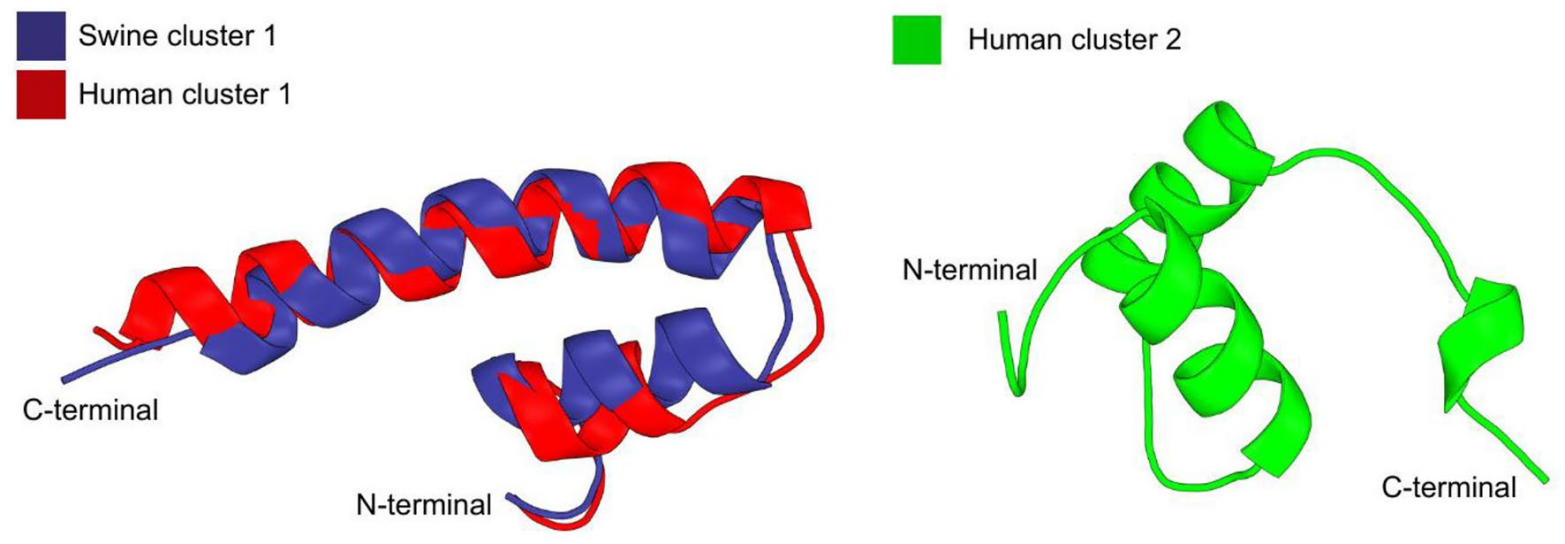
Predicted tridimensional structures of protein clusters modeled using AlphaFold. Two clusters from human sequences (Human cluster 1 and 2) and one cluster from swine (Swine cluster 1) were generated from consensus sequences with PyMOL

Given the importance of heat-labile enterotoxin in disease development, we compared the sequence and structural features of STb and STa in the analyzed ETEC strains. The swine-exclusive PF09075 domain comprises the only domain found in STb (UniProt: P22542; 71 amino acid), spanning residues 24 to 71. DIAMOND BLASTp searches against swine and human datasets confirmed that this protein was consistently detected only in swine-derived sequences, even when coverage and identity thresholds were reduced to 40% and 20%, respectively, suggesting strong host specificity. We performed similarity analysis of STb with STa (UniProt: P01559), another heat-stable enterotoxin that contains the PF02048 domain, which is found in both human- and swine-derived sequences (Fig. 5). This enabled a comparative assessment of structural and sequence features between STa and STb, aiming to identify potential differences that may contribute to host adaptation and pathogenicity.

Due to the absence of homologous sequences in human-associated ETEC, we compared the sequences of STa enterotoxin (UniProt ID: P01559), which contains the PF02048 domain, shared by both human- (32 sequences) and swine-derived (19 sequences) ETEC included in this study. For human-derived ETEC, the consensus sequence analysis was of STa (24–71 aa, Supplementary Fig. 1A) identified two clusters: (i) Human cluster 1: SLDSSKEKITLETKKCDVVKNNSEKKSENMNNTFYCCELCCNPACAGC, comprised by four sequences with 87.5–100% identity; and (ii) Human cluster 2: PVESSKEKITLESKKCNIAKKSNKSGPESMNSSNYCCELCCNPACTGC, comprised by 28 sequences with 85.4–100% identity. The same procedure was applied to analyze the 19 sequences of swine-derived ETEC (24–71 aa, Supplementary Fig. 1B). In this case, only one cluster was found: Swine cluster 1: SLDSSKEKITLETKKCDVVKNNSEKKSENMNNTFYCCELCCNPACAGC, with 100% identity. The alignment revealed a high degree of structural conservation, consistent with their high sequence identity, with a root mean square deviation (RMSD) of 1.50 Å for the backbone atoms.

Comparative analysis between human- and swine-derived clusters and determined Human cluster 1 shares 100% identity with Swine cluster 1, indicating complete conservation of the STa sequence between this subset of ETEC strains; whereas Human cluster 2 has 60.4% identity with both Human cluster 1 and Swine cluster 1 (Supplementary Fig. 1C). Considering the function domain region of STa (60–71 aa), the only difference between Human cluster 2 (CCELCCNPACTGC, PDB ID: 7D37) and both Human cluster 1 and Swine cluster 1 (CCELCCNPACAGC, predicted by AlphaFold: AF-A0A193M1P5-F1-v4) is a threonine-to-alanine substitution at position 69, which removes a polar hydroxyl group and may alter local hydrogen bonding, potentially affecting the structure and stability of the C-terminal tail (Fig. 3).

## Discussion

ETEC infection depends directly on the presence of specific receptors in the host gut for colonization, followed by the production of enterotoxigenic toxins that interact with host receptors to trigger disease. While microbial adherence is a key contributor to the ETEC host specificity, other factors such as the strain’s ability to survive and replicate within the host, as well as mechanisms for acquiring metal ions from the environment, are also important features potentially linked to host adaptation (Baumler and Fang 2013). Other relevant factors include genomic decay, genome arrangements, and horizontal gene transfer (Baumler and Fang 2013). Therefore, pangenomic tools are valuable for understanding of bacterial diversity and genome structure dynamics.

In this study, three main findings emerged from our comparative genomic analyses. First, phylogenetic analysis showed that ETEC strains cluster primarily according to sequence type (ST), rather than host species, with evidence of shared STs between humans and swine. Second, although the core functional repertoire is largely conserved across hosts, the presence of host-exclusive domains, based on the analyzed dataset, suggests differential adaptative strategies for each one. Third, enterotoxin analysis revealed a strong association of STb with swine-derived ETEC and greater sequence variability of STa among human-derived strains, while some STa variants were shared between hosts, suggesting partial evolutionary overlap.

Using pangenomic tools, we explored the evolutionary paths of ETEC strains across different hosts. The open and dynamic structure of ETEC genomes suggest a high degree of genome plasticity, where the continuous acquisition of accessory genes facilitates rapid adaptation to diverse niches and selective pressure. Moreover, to assess the genetic variability within the analyzed dataset, we performed a SNP distribution analysis based on the core genomes of human- and swine-derived ETEC, which revealed a high genetic variability among the analyzed genomes, particularly in human-derived ETEC. Several clusters were associated with specific STs, but we also observed the presence of different STs within the same subclade, indicating potential diversification within closely related genomes. In addition, no strict geographical clustering was observed, as genomes from different locations were distributed across multiple clades. However, the limited geographical diversity of the swine-derived ETEC strains, most of which were isolated in Belgium, restricts the interpretation of this dataset.

According to our analysis, the most frequently reported ETEC sequence types to date are *E. coli* ST10 (13%), ST443 (9%) and ST772 (5%). Although our data indicates a degree of host specificity in ST distribution, the limited number of ETEC genomes currently available in public databases constrains the strength of this interpretation. Furthermore, the geographic distribution of the analyzed genomes was not homogeneous between host groups, as human-derived ETEC were isolated in a broader range of regions, mainly in Asia and Africa, whereas swine-derived ETEC originated mainly from Europe. This uneven geographic representation might be a potential bias, and some of the observed host-associates differences could partially reflect regional variation rather than host-specific adaptation. In addition, we observed that *E. coli* ST10 and ST4 have been identified in clinical samples from both human and swine hosts, which may indicate a potential epidemiological link between ETEC strains circulating in veterinary and human infections. Enterotoxigenic *E. coli* ST10 has been commonly described in diarrheic piglets (Jiang et al. 2019; García et al. 2020), however a previous study showed its occurrence in both humans and animals, suggesting the potential ability of *E. coli* ST10 to cross the host barrier (Shabana et al. 2013). Moreover, phylogenetic analysis indicates that ETEC strains tend to cluster primarily based on their ST, regardless of host species or geographical origin. The use of housekeeping genes in MLST analysis is considered the gold standard for epidemiological studies, as these genes are constitutive and conserved across all strains of the same species, thus forming part of the core genome. Interestingly, even the ETEC accessory genome, which consists of less conserved regions enriched with horizontally acquired genes, also grouped the analyzed strains mostly by ST, reinforcing the strong evolutionary signal associates with ST rather than host origin.

The search for functional domains demonstrated that most ETEC domains are shared across human- and swine-derived strains, and are part of the most frequent occurring domains in ETEC, suggesting that major functional roles are preserved regardless the host species. Still, the existence of host-exclusive domains implies slightly functional differences between human- and swine-derived strains. Our findings indicated that human-derived ETEC have exclusive domains potentially related to adhesion, transposition, and toxins, which may enhance host colonization and genome plasticity in the human gut. In addition, the enrichment in transposition-related domains may suggest a more dynamic repertoire of mobile genetic elements in human-derived ETEC strains, indicating that horizontal gene transfer plays an important role in bacterial adaptation. This increased genomic plasticity may facilitate the acquisition of colonization and virulence factors, contributing to ETEC persistence in the host.

In turn, swine-derived ETEC have exclusive domains mainly associated with stress response and metal binding, which may reflect adaptation to environmental and physiological conditions specific to the swine gut, including nutrient availability and stress tolerance. The swine gut environment, influenced by diet composition, growth rates, and intensive farming practices, may impose distinct selective pressures such as oxidative stress, and fluctuating metal availability, such as iron, zinc, or copper feed supplementation. Therefore, the enrichment of metal-binding and stress-response domains could enhance bacterial fitness under conditions of metal or redox imbalances, which ma. Such adaptations may improve persistence and competitiveness within swine gut.

Interestingly, according to our data, PF09075 domain from STb protein has only been detected in swine-derived ETEC strains. Although past studies have described ETEC-STb in humans (Lortie et al. 1991; Okamoto et al. 1993), currently, STb is considered primarily associated with swine-derived ETEC strains (Duan et al. 2012). In accordance, our findings and to the best of our knowledge, no human-derived STb was predicted in the evaluated genomes, which suggest that ETEC harboring this heat-labile enterotoxin may exhibit a tropism for the swine host.

In addition, given the importance of heat-labile enterotoxins STa and STb, we compared the domains contained in these proteins (STa-PF02048 and STb-PF09075) between human- and swine-derived strains, in order to better understand whether sequence or structural differences could contribute to host tropism. Notably, STb and STa shares no homology at structural or functional level (Dubreuil 2008); however, both proteins play crucial roles in the pathogenesis of ETEC infections. STa, in particular, is classified based on the host from which ETEC was isolated, including STh (produced solely in ETEC from humans) and STp (in pigs, bovine, and humans) (Nair and Takeda 1998). Hence, we compared STa proteins from the 32 human- and 19 swine-derived ETEC included in this study, and determined two ETEC clusters for human-derived ETEC and one cluster for swine-derived ETEC. Such results indicate higher variability of enterotoxin I (STa) in human-derived ETEC in comparison to ETEC isolated from swine, corroborating previous study that showed no heterogeneity in STa among ETEC isolated from diarrheal pigs based on gene sequencing (Zhang et al. 2009). Additionally, STa Human cluster 1 was identical to Swine cluster 1, reinforcing our earlier observation that certain ETEC strains circulating in humans and swine may be closely related or shares a common evolutionary origin. Altogether, our findings suggest that heat-labile enterotoxins STa and STb are highly conserved in swine-derived ETEC strains, while human-derived strains exhibit greater STa variability, potentially reflecting distinct selective pressures or broader evolutionary differentiation.

These findings suggest that the genetic barrier for cross-host transmission between humans and swine is limited for certain ETEC STs, supporting the possibility of zoonotic or reverse zoonotic exchange. However, the presence of host-specific functional domains and the strong association of STb with swine-derived ETEC indicate that host-specific adaptations may contribute to bacterial host tropism. Therefore, although cross-host transmission appears biologically plausible, functional genomic differences may still contribute to maintaining a partial barrier to successful colonization and host-adaptation.

## Conclusion

This comprehensive pangenomic comparison and functional analysis of human- and swine-derived ETEC suggest that although the presence of ETEC ST10 and ST4 indicates a potential epidemiological link between strains involved in veterinary and human infections, functional differences between human- and swine-derived strains contribute to host specificity. In particular, sequence and functional differences in the heat-labile enterotoxins STa and STb appear to drive host tropism of ETEC strains. Altogether, these findings suggest that while certain ETEC strains may circulate across hosts, host-specific functional adaptations potentially contribute to niche adaptation and maintenance of host association.

## Supplementary Information

Below is the link to the electronic supplementary material.

Supplementary file1 (PDF 792 KB)

Supplementary file2 (XLSX 86 KB)

Supplementary file3 (XLSX 12 KB)

Supplementary file4 (XLSX 39 KB)

## Data Availability

The data underlying this article are publicly available at the National Center for Biotechnology Information (NCBI) [URL: https://www.ncbi.nlm.nih.gov/datasets/genome/].
