# Supplementary figures and images for: Pangenomic and functional domain comparison of enterotoxigenic Escherichia coli isolated from humans and swine: insights into host specificity
Source: Mol Genet Genomics. 2026 Apr 24;301(1):98. doi: 10.1007/s00438-026-02412-4 (PMC13109261; doi:10.1007/s00438-026-02412-4)

A

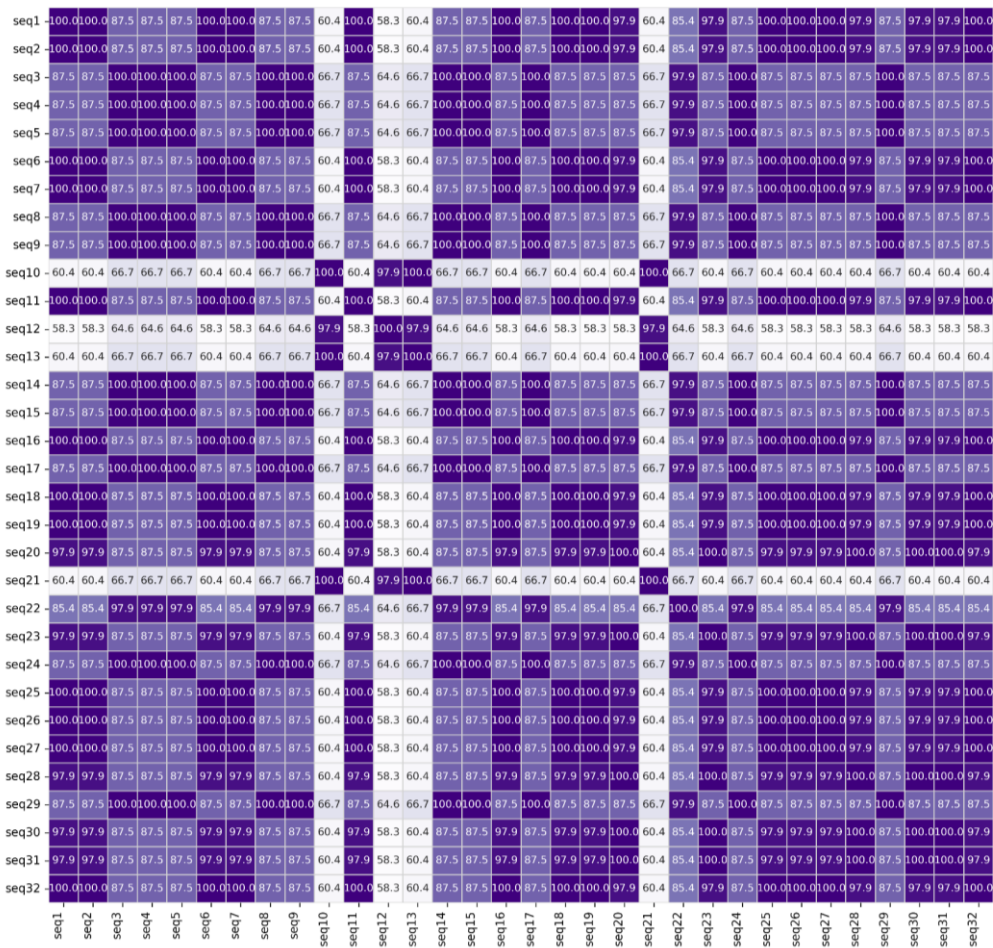

B

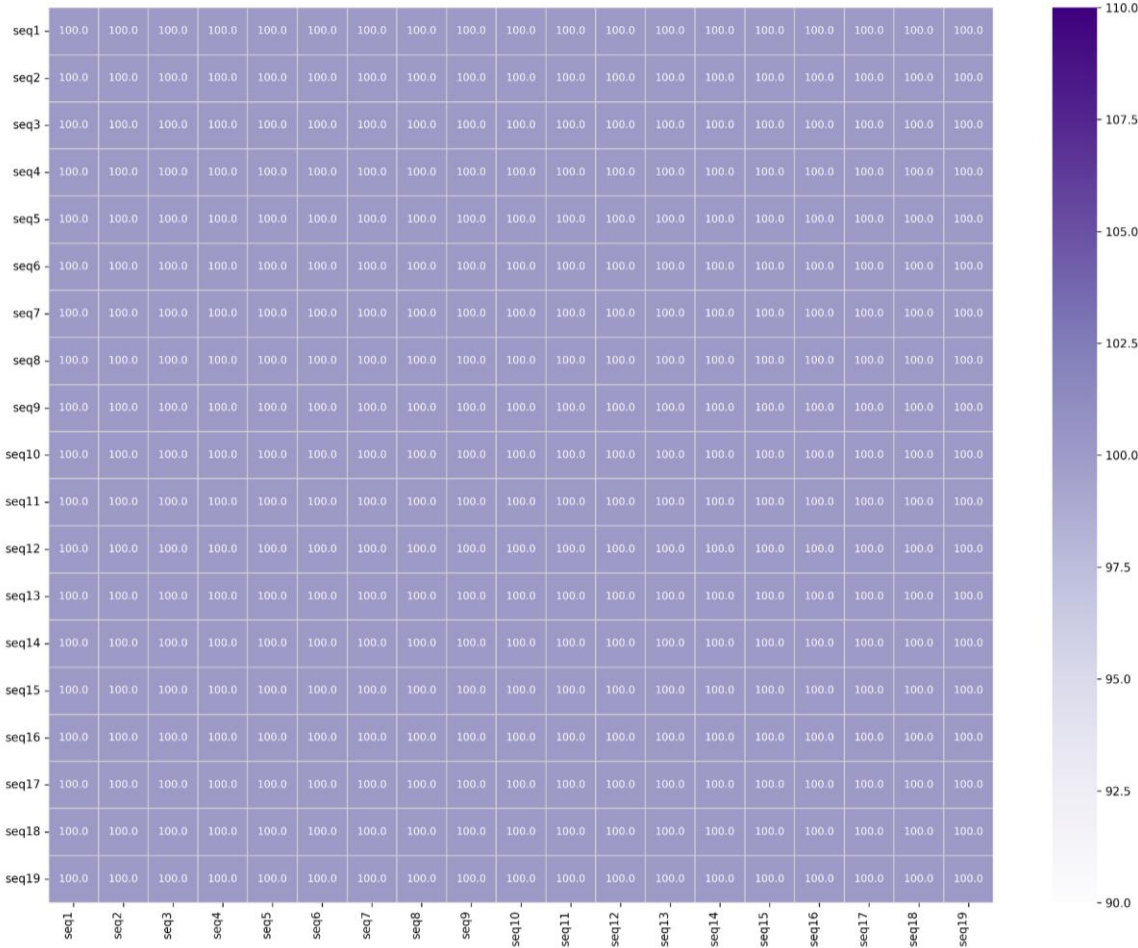

C

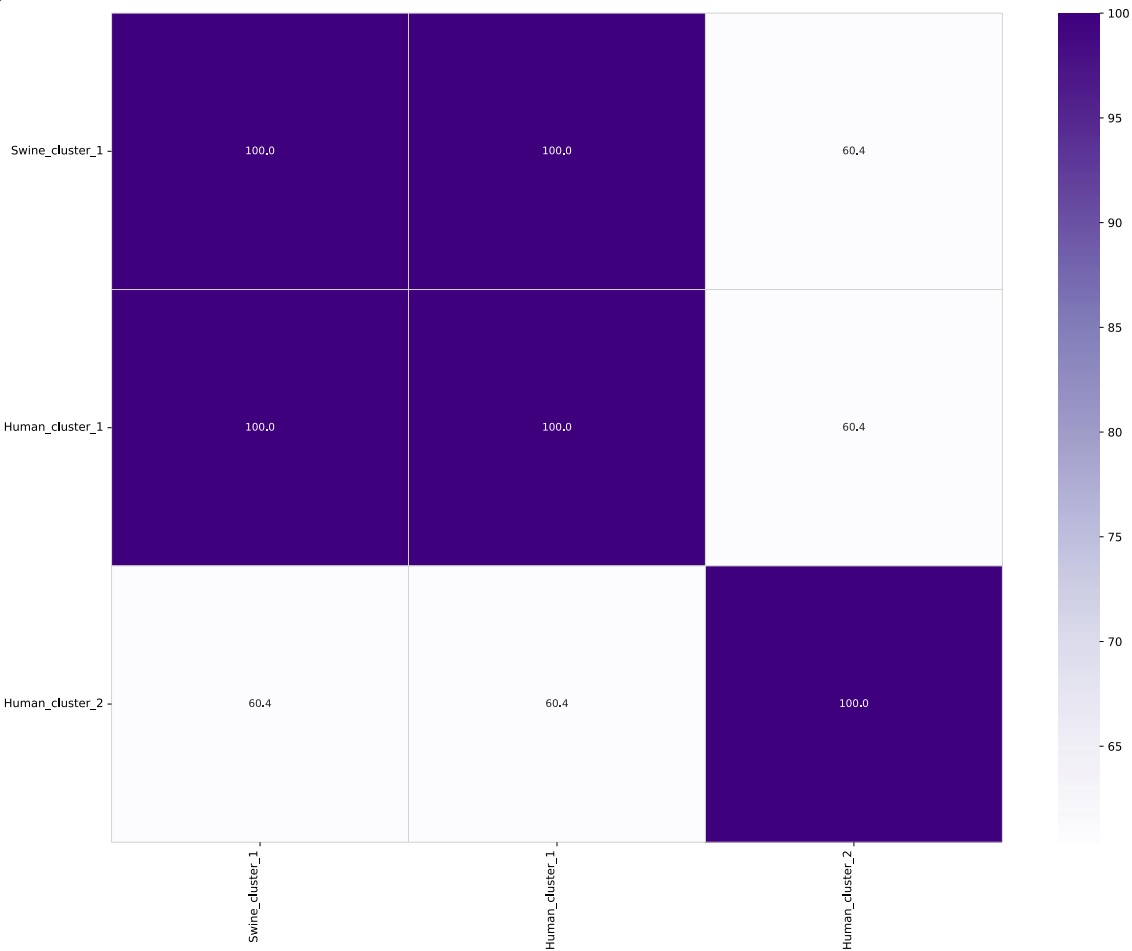

Supplement: Supplementary file 1 — Supplementary file1 (PDF 792 KB) [file 438_2026_2412_MOESM1_ESM.pdf]
